# A case report: the use of ultrasound guided peripheral nerve block during above knee amputation in a severely cardiovascular compromised patient who required continuous anticoagulation

**DOI:** 10.1097/MD.0000000000009374

**Published:** 2018-03-02

**Authors:** Myong-Hwan Karm, Sohee Lee, Syn-Hae Yoon, Sukyung Lee, Wonuk Koh

**Affiliations:** a Department of Dental Anesthesiology, Seoul National University Dental Hospital; b Department of Anesthesiology and Pain Medicine, Asan Medical Center, University of Ulsan College of Medicine, Seoul, Republic of Korea.

**Keywords:** amputation, anticoagulation, case report, nerve block, ultrasound

## Abstract

**Rationale:**

Although lower–extremity surgeries are mainly performed under general or central neuraxial anesthesia, ultrasound-guided peripheral nerve block (PNB) can be a good alternative, especially for patients who require continuous anticoagulation treatment and present with poor cardiovascular conditions.

**Patients concerns:**

The patient required continuous anticoagulation treatment due to the high risk of thromboembolism and poor cardiovascular conditions.

**Diagnoses:**

The patient required lower-extremity amputation due to atherosclerotic and thromboembolic obstruction.

**Interventions:**

We decided to progress the operation under peripheral nerve block. The sciatic, femoral, lateral femoral cutaneous nerve and anterior branch of the obturator nerve were blocked under ultrasound guidance. Sixty ml of a local anesthetic (equal mix of 1% lidocaine and 0.375% ropivacaine) was administered for the block.

**Outcomes:**

Sufficient sensory block at the area of amputation was obtained, and the patient's vital signs were stable throughout surgery.

**Lessons:**

Ultrasound-guided peripheral nerve block can be an excellent anesthetic technique for patients receiving radical surgery at the proximal lower extremities, especially patients who are critically ill and considered high risk.

## 1 Introduction

Choosing the optimal anesthetic technique is a challenge for anesthesiologists and pain physicians in everyday surgical theaters. Anesthesiologists and pain physicians always try to choose the appropriate anesthetic technique after taking into account surgical requirements, operating room status, patient medical conditions, patient preferences, and technical skill. Although lower-extremity surgeries are mainly performed under general or central neuraxial anesthesia,^[1,2]^ ultrasound-guided peripheral nerve block (PNB) can be a good alternative,^[3]^ especially for patients who require continuous anticoagulation treatment and present with poor cardiovascular conditions.^[4]^ Combination of femoral and sciatic nerve block is reportedly an effective anesthetic technique for outpatient knee surgery,^[5,6]^ and is also known to be an effective method for postoperative analgesia in patient undergoing major lower limb operations.^[7]^ But few cases of lower limb amputation under PNB have been reported. Furthermore, because of the large amount of required local anesthesia, above-knee amputation may present with a higher risk of systemic local anesthetic toxicity compared with the amputation below the knee.

In the case presented here, our patient required lower-extremity amputation due to atherosclerotic and thromboembolic obstruction. The patient also required continuous anticoagulation treatment due to the high risk of thromboembolism and presented with poor cardiovascular conditions. Because of the possible risk of hemodynamic instability and epidural hematoma following the administration of general or central neuraxial anesthesia, we chose ultrasound-guided PNB for the anesthetic technique to achieve an acceptable level of anesthesia with minimal hemodynamic disturbance.

## 2 Case report

### 2.1 Patient characteristics

A 61-year-old female patient (154 cm; 59 kg; American Society of Anesthesiologists physical status IV) was admitted with symptoms of dysarthria and global aphasia. The patient was diagnosed with multifocal embolic cerebral infarction (right basal ganglia, left frontal lobe), left common iliac artery occlusion, and right popliteal artery embolic occlusion. Both legs demonstrated acute ischemia. The patient had a history of moderate rheumatic mitral stenosis and regurgitation, paroxysmal atrial fibrillation, and congestive heart failure. Transthoracic echocardiography confirmed moderate mitral stenosis and regurgitation with multiple left atrial thrombi, akinesia in the left ventricular (LV) apex, anteroseptum with severe LV dysfunction (27% LV ejection fraction), and moderate pulmonary hypertension with right ventricular dysfunction. Cardioversion was performed on admission because of paroxysmal atrial fibrillation, and intravenous (IV) heparinization was initiated as part of anticoagulation treatment. After the start of anticoagulation, right leg ischemia improved but left leg ischemia worsened due to the formation of gangrene below the knee joint. The patient was referred to the orthopedic surgeon for acute lower limb ischemia, and below-knee amputation with optional above-knee amputation was decided. Anticolagulation (IV heparinization) was continued because the patient was at high risk of thromboembolism, and the last activated prothrombin time determined before surgery was 47.4 seconds. The international normalized ratio was 1.44. After considering these conditions, we decided to perform the operation under ultrasound-guided PNB rather than general or central neuraxial anesthesia. In order to achieve sufficient anesthesia for above-knee amputation, we planned to block the sciatic, femoral, and lateral femoral cutaneous nerve, and the anterior branch of the obturator nerve.

### 2.2 Regional anesthesia technique

**Figure 1:**
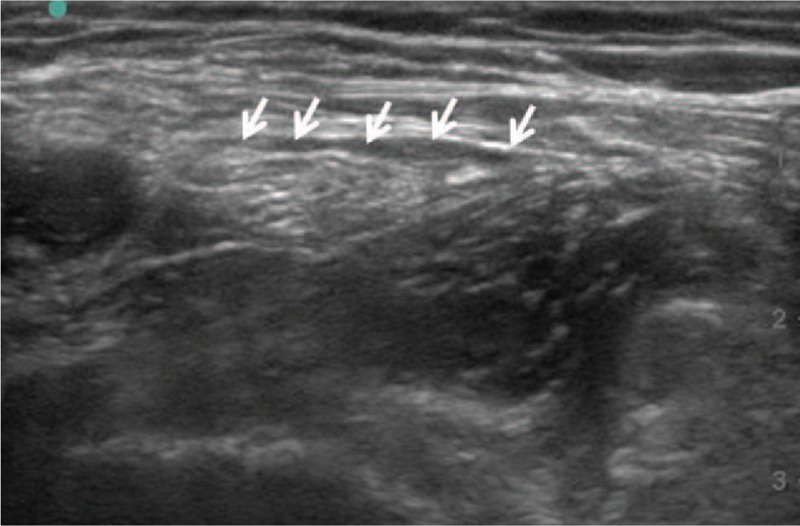
Ultrasound image of the femoral nerve. The local anesthetic was deposited between the fascia iliac and femoral nerve (arrows).

**Figure 2:**
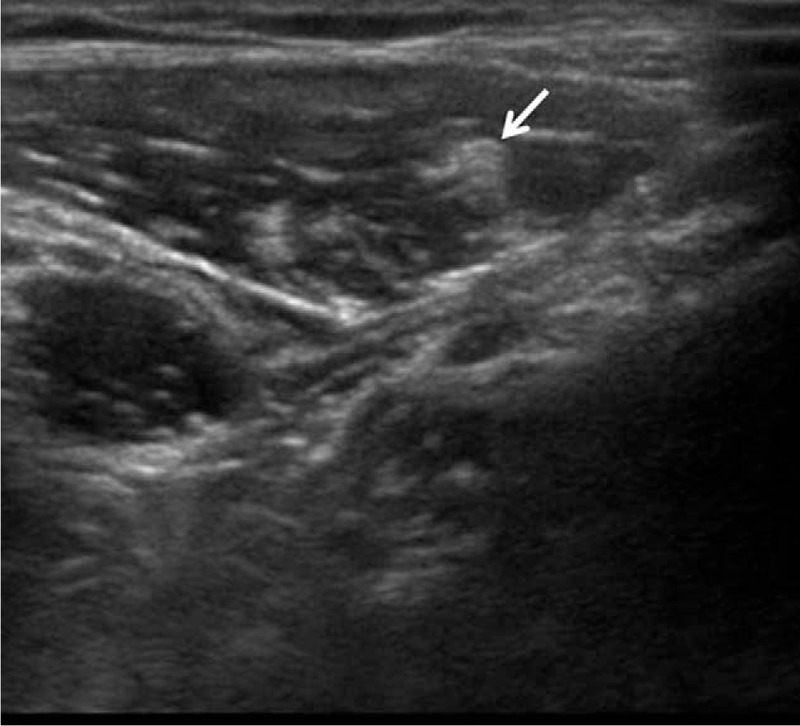
Ultrasound image of the lateral femoral cutaneous nerve (arrow).

**Figure 3:**
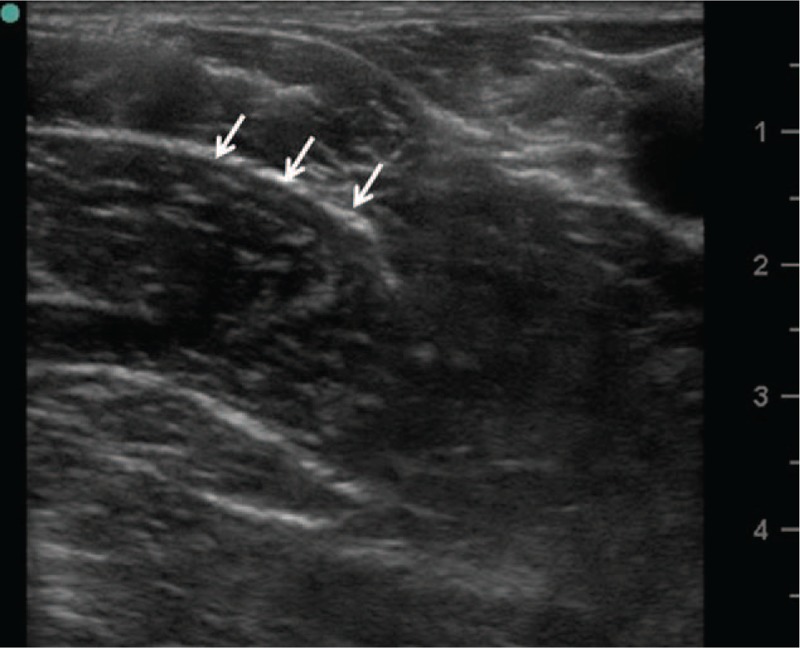
Ultrasound image of the anterior branch of the obturator nerve (arrows).

**Figure 4:**
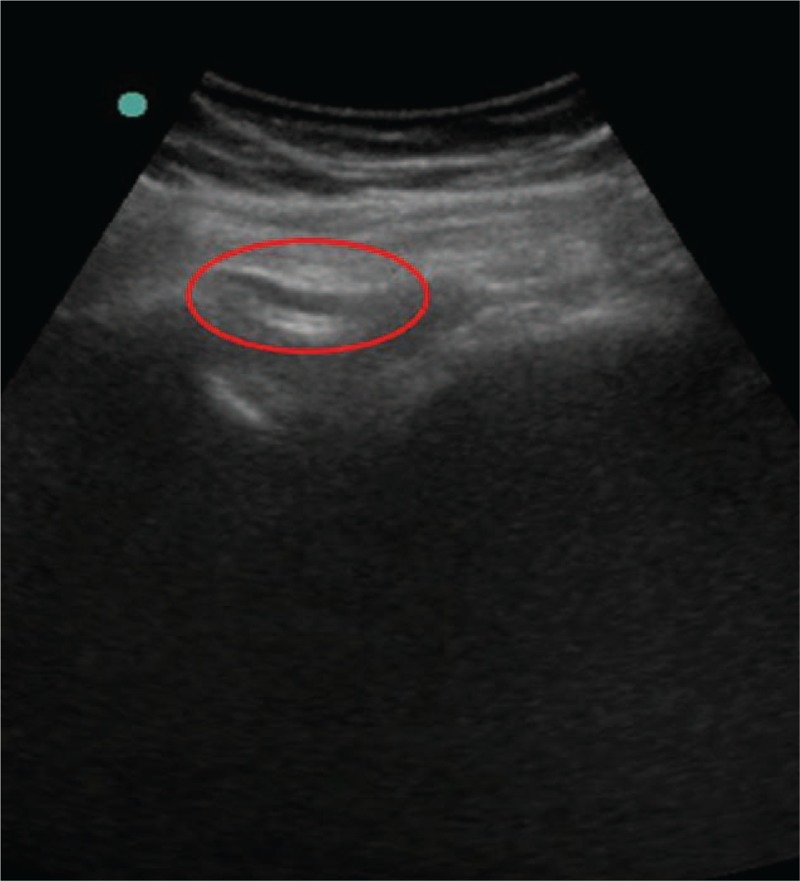
Ultrasound image of the sciatic nerve. The local anesthetic is deposited under the gluteus maximus muscle, and the sciatic nerve is highlighted (circle).

The patient presented with an initial blood pressure of 150/85 mmHg, heart rate of 88 beats/min, and 98% oxygen saturation upon arrival at the operating room. The patient was slightly drowsy, but could understand simple commands. The patient was placed in the supine position, and the thigh was slightly abducted and externally rotated. Standard monitoring was performed (eg, electrocardiography, pulse oximetry, and noninvasive blood pressure), and 4 L/min oxygen was delivered via facial mask. A 15 to 6 MHz high-frequency linear array transducer (HGL50x; SonoSite, Bothell, WA) and ultrasound (S–Nerve; SonoSite) was used to trace the femoral, lateral femoral cutaneous, and obturator nerves (Figs. 1–3). For sciatic nerve blockage, the patient was laterally positioned the affected thigh and knee were flexed, and the sciatic nerve was traced using a 5 to 2 MHz low-frequency curved array transducer (C60x; Sonosite) (Fig. 4). Using real-time ultrasound guidance, a 22-gauge 60 mm stimulating needle (for femoral, lateral femoral cutaneous, and obturator nerve block) and 22-gauge 100 mm stimulating needle (for sciatic nerve block) (StimuplexD; B.Braun AG, Melsungen, Germany) were advanced within the proximity of each targeted nerve. The nerve stimulator (MultiStim SENSOR; PAJUNKGmbH Medizintechnologie, Geisingen, Germany) was used to localize the nerves with fade of electrical currents at 0.5 mA and twitch the corresponding muscles (ie, the hamstring for the sciatic nerve, quadriceps for the femoral nerve, and the adductor muscles of the thigh for the obturator nerve).

A 60 mL volume of local anesthetic (equal mix of 1% lidocaine and 0.375% ropivacaine) was slowly injected with intermittent aspiration after final confirmation. A sciatic nerve block (25 mL) was administered via the transgluteal approach, a femoral nerve block (20 mL) was administered just under the inguinal fossa, a lateral femoral cutaneous nerve block (7 mL) was administered at the origin of sartorius, and the anterior branch of the obturator nerve (8 mL) was blocked between the adductor brevis and longus. Below-knee amputation was initially performed, and the patient obtained sufficient sensory block at the area of amputation without further analgesia. After below-knee amputation, above-knee amputation was decided by the surgeon due to poor stump condition. The patient developed little pain during incision of the medial thigh and was managed using adjuvant IV opioids (2 μg/kg fentanyl). The surgery required 2 hours and vital signs were stable throughout the operation.

### 2.3 Ischemic insults

At the end of surgery, the patient suddenly became irritable and only responded to painful stimuli. The patient status was notified to the surgeons and prompt neurologic and radiologic examination was recommended. Emergency brain computed tomography and diffusion magnetic resonance imaging were immediately performed after the operation. The magnetic resonance imaging images showed no significant differences in comparison with previously obtained images, but computed tomography suggested more prominent attenuation in previously infarcted areas. Transient ischemic attack was suspected, and immediate thrombolytic treatment was initiated in the intensive care unit. The patient became drowsy within 1 day after surgery, and 2 days after surgery, the patient was alert but aphasia remained. The patient remained in the hospital for 2 months to manage heart failure and infectious conditions, and was discharged after her conditions improved.

## 3 Discussion

When managing critically ill and hemodynamically unstable patients, choosing a reliable anesthetic technique that result in minimal hemodynamic effects is crucial. The current case report demonstrates that ultrasound-guided PNB may be suitable for radical surgery of the lower extremities in severely hemodynamically compromised patients. Ultrasound-guided PNB can provide perioperative hemodynamic stability to patients with known to have poor cardiovascular conditions. Furthermore, ultrasound guidance enables the anesthesiologist to visualize the vascular structures, which makes this technique feasible for patients with coagulopathies and those receiving anticoagulation.

According to a propensity score-matched observational study, the 30 day mortality is higher among patients who receive major lower-extremity amputations under general anesthesia in comparison with regional anesthesia (central neuraxial and PNB).^[8]^ PNB is reportedly associated with better hemodynamic stability and shorter hospital stays in comparison with general anesthesia among elderly patients with hip fracture.^[9]^ Moreover, PNB is associated with better functional recovery and superior postoperative pain control.^[10,11]^ It is also associated with a reduced risk of acute postoperative confusion and deep venous thrombosis.^[12]^

Compared with central neuraxial anesthesia, PNB demonstrates several advantages. PNB minimizes pruritus, urinary retention, and hypotension, and reduces the risk of spinal hematoma and infection. In addition, patients with antiplatelet or anticoagulant therapy can undergo certain PNB procedures without significant risk.^[13]^ However, PNB also demonstrates several complications. Including direct nerve trauma, incomplete nerve blockade, infection, local hematoma, ischemic injury, and requires the systemic IV injection of local anesthetic agents.^[13]^

The use of ultrasound guidance demonstrates several advantages over landmark-guided PNB techniques with or without nerve stimulation.^[14]^ Ultrasound can visualize the target and adjacent neural anatomical structures including vascular structures. In addition, ultrasound allows the use of smaller volumes of local anesthetics. Ultrasound-guided PNB also demonstrates faster onset time, shorter block performance time, and a higher block success rate.^[15]^

Considering the advantages of PNB in comparison with general and neuraxial anesthetic techniques, we decided to use ultrasound-guided PNB for above-knee amputation in our patient who required continuous anticoagulation treatment and presented with poor cardiovascular conditions; ultrasound-guided PNB subsequently demonstrated satisfactory anesthesia. We performed nerve blocks to the sciatic, femoral, lateral femoral cutaneous nerves, and anterior branch of the obturator nerve. However, the patient demonstrated painful responses during dissection of the medial thigh immediately above the knee capsule, and this may have been due to the incomplete blocking of the posterior branch of the obturator nerve, which mainly innervates the adductor magnus muscle.^[16]^ Blocking both the anterior and posterior branches may have improved the satisfactory rate, but this was not performed because of the risk of local anesthetic overdose. In fact, total dose of local anesthetic used was above the recommended doses.^[17]^ However, there is not any systemic toxicity and the nerve blocks were all performed without intravascular punctures. We performed PNB by recommendations for preventing local anesthetic systemic toxicity.^[18]^

In conclusion, radical surgery of the proximal lower-extremities can be safely performed on high-risk patients with minimal hemodynamic disturbance under ultrasound-guided PNB. Furthermore, in patients at high risk of thromboembolism, like our current patient subject, prompt detection of the event during surgery is possible. Therefore, ultrasound-guided PNB is an excellent anesthetic technique, and especially useful for treating critically ill, high-risk patients. Further studies are needed to evaluate the perioperative outcomes and complications of ultrasound-guided PNB for radical proximal lower-extremity surgery.
